# Paternal and maternal exposures to per- and polyfluoroalkyl substances (PFAS) and birth outcomes: a multi-country cohort study

**DOI:** 10.1186/s12940-025-01199-y

**Published:** 2025-07-12

**Authors:** Pengfei Guo, Jiajun Luo, Jie Zhang, Jens Peter Bonde, Paweł Struciński, Viktor Ohniev, Onyebuchi A. Arah, Nicole C. Deziel, Joshua L. Warren, Gunnar Toft, Zeyan Liew

**Affiliations:** 1 Department of Environmental Health Sciences, Yale School of Public Health, 60 College Street, CT 06510 New Haven, USA; 2 Yale Center for Perinatal, Pediatric, and Environmental Epidemiology, Yale School of Public Health, New Haven, CT USA; 3 Department of Surgery, Biological Sciences Division, University of Chicago, Chicago, IL USA; 4 Institute for Population and Precision Health, University of Chicago, Chicago, IL USA; 5 Steno Diabetes Center Aarhus, Aarhus University Hospital, Aarhus, Denmark; 6 Department of Occupational and Environmental Medicine, Bispebjerg-Frederiksberg University Hospital, Copenhagen, Denmark; 7 Department of Toxicology and Health Risk Assessment, National Institute of Public Health, NIH - National Research Institute, Warsaw, Poland; 8 Department of Public Health and Healthcare Management, Kharkiv National Medical University, Kharkiv, Ukraine; 9 Department of Epidemiology, Fielding School of Public Health, University of California, Los Angeles (UCLA), Los Angeles, CA USA; 10 Department of Statistics and Data Science, UCLA College, Los Angeles, CA USA; 11 Practical Causal Inference Lab, UCLA, Los Angeles, CA USA; 12 Research Unit for Epidemiology, Department of Public Health, Aarhus University, Aarhus, Denmark; 13 Department of Biostatistics, Yale School of Public Health, New Haven, CT USA

**Keywords:** Environmental pollutants, Endocrine disruptors, PFAS, Paternal exposure, Pregnancy, Birth outcome

## Abstract

**Background:**

Maternal prenatal exposures to per- and polyfluoroalkyl substances (PFAS) have been linked to adverse birth outcomes. However, few investigations have considered paternal PFAS exposure. We estimated the parent-specific associations of prenatal PFAS exposures with adverse birth outcomes.

**Methods:**

This study included 498 couples from the INUENDO cohort recruited at antenatal care visits in Greenland, Poland, and Ukraine during 2002–2004. We measured five major types of PFAS in parental serum during pregnancy. We analyzed three birth outcomes ascertained from medical records, including gestational age, birth weight, and birth length. We used weighted least squares linear regression to evaluate parent-specific associations of serum PFAS with the birth outcomes, adjusting for parental co-exposures and covariates. We also used quantile g-computation for mixture modeling of the birth outcomes of paternal and/or maternal exposures to multiple PFAS.

**Results:**

No associations were found between maternal and paternal PFAS exposures and gestational age. However, after adjusting for paternal PFOA, a higher level of maternal serum perfluorooctanoate (PFOA) was linked to a tendency towards lower birth weight and shorter birth length. Paternal exposure to several PFAS was also associated with a tendency for shorter birth length, but the estimated effect sizes were small. We found no joint exposure effects in the mixture analyses.

**Conclusions:**

While the evidence was inconclusive, maternal PFOA and paternal PFAS exposures seemed to be associated with lower offspring birth weight and shorter birth length, respectively. Parent-specific effects of PFAS exposures on offspring growth and development warrant further research.

**Supplementary Information:**

The online version contains supplementary material available at 10.1186/s12940-025-01199-y.

## Introduction

Mounting evidence demonstrates that maternal prenatal and intrauterine environmental exposures can adversely affect birth outcomes and offspring health [1–6]. While less research has focused on paternal factors, paternal advanced age [7, 8], preconception occupational exposure [9, 10], cigarette smoking [11–13], alcohol intake [14–16], obesity, and diet [15, 17] have also been associated with offspring-specific birth defects or childhood asthma. Growing mechanistic studies have also suggested that paternal exposure to environmental chemicals could adversely impact semen quality and offspring development through changes in the sperm epigenome [18–21]. Still, the epidemiological associations between paternal exposure to environmental pollutants and fetal development have been studied sporadically [21, 22].

Ubiquitous and emerging endocrine-disrupting chemicals (EDCs) pose exposure health threats to parents and their offspring globally [23, 24]. Perfluoroalkyl and polyfluoroalkyl substances (PFAS) are among the most concerning EDCs linked to metabolic alterations [25] and disorders [26] with diverse industrial and commercial applications, such as non-stick cookware, cosmetics, fire-fighting foams, and food packaging [27]. The long-chain PFAS are highly persistent in the environment with an average human half-life of 4–8 years [28, 29]. PFAS can cross the human placental barrier [30], and maternal prenatal exposure to several PFAS has been repeatedly linked to impaired fetal growth in pregnancy cohort studies conducted across populations [31–33]. In contrast, only three cohort studies from the United States (US) and China have investigated whether paternal PFAS exposure may influence fetal development [34–36]. Two previous studies reported null associations between paternal preconception or prenatal PFAS exposure on offspring birth weight [34, 35], while one study reported a positive association [36]. Additional studies that investigate paternal exposure effects on offspring health are needed. Moreover, a parental comparative analysis within the same cohort may offer insights into paternal-specific exposure effects and the impact of confounding factors shared among the parents [37–39].

Here, we studied the parental prenatal serum-PFAS levels from the multi-country INUENDO birth cohort and their associations with offspring gestational age, birth weight, and birth length [40, 41]. Previously, a study from the INUENDO cohort reported that maternal prenatal serum PFOA was associated with a lower birth weight among term births [42]. Still, the study did not consider paternal PFAS exposure or evaluate other birth outcomes. We utilized mixture modeling methods to estimate the potential joint effects of paternal or maternal exposures to multiple PFAS on birth outcomes [24].

## Methods

### Study design and population

Our study was based on a multi-country longitudinal birth cohort, INUENDO (Biopersistent organochlorines in diet and human fertility), a European Union Fifth Framework Program Research and Development project. Details of the cohort design and data collection have been described elsewhere [43–45]. Briefly, the INUENDO cohort recruited parents (pregnant women and their male spouses) who attended antenatal care visits between May 2002 and February 2004 at one of three study regions: (1) local hospitals in 19 municipalities and settlements throughout Greenland; (2) at a large central hospital in Warsaw (Poland); and (3) at three hospitals and eight antenatal clinics in Kharkiv (Ukraine).

Parents who were born in the country of study and aged at least 18 years at enrollment were eligible. Parents were asked to each provide a venous blood sample during pregnancy and participate in an interview at the clinic which collected data on socioeconomic factors, lifestyles, and medical history. Figure S1shows the flow diagram illustrating the inclusion and exclusion of study participants. This study included 498 liveborn singleton pregnancies from Greenland (*n* = 178), Poland (*n* = 142), and Ukraine (*n* = 178), for which serum samples from both parents were available for PFAS measurements, and childbirth outcomes (including newborn sex) were accessible for statistical analyses. One birth from Ukraine was excluded due to birth weight and birth length being more than five standard deviations below the mean.

As quality control procedures, both paternal and maternal questionnaires were translated into native language in the participating countries and back translated to English for error checking. All questionnaires were centrally double entered, and inconsistencies were resolved by looking up the original data if necessary. All interviews as well as collection, processing, storage and shipment of blood samples were performed under uniform research protocols across study sites. The study was approved by the local ethics committee in each of the participating countries. Written consent was obtained from all participating parents.

### PFAS exposure assessment

Paternal and maternal blood samples were collected during mid- to late-pregnancy (median of gestational weeks of blood draw in this study: 30) for biochemical measurements. PFAS measurements were conducted as part of the CLEAR (Climate change, environmental contaminants and reproductive health) project at The Department of Occupational and Environmental Medicine in Lund, Sweden. Seven PFAS analytes were measured in 100-µl serum aliquots by liquid chromatography tandem mass spectrometry (LC/MS/MS) [46], including perfluorooctanesulfonic acid (PFOS), perfluorooctanoate (PFOA), perfluorononanoic acid (PFNA), perfluorodecanoic acid (PFDA), perfluorohexanesulfonic acid (PFHxS), perfluoroundecanoic acid (PFUnDA), and perfluorododecanoic acid (PFDoDA).

This study included five PFAS that exceeded the limit of detection (LOD) in > 80% of the study population (PFOS, PFOA, PFNA, PFDA, and PFHxS). LOD was defined as the concentrations corresponding to three times the standard deviation of the responses in chemical blanks and was published previously [46, 47]. Values below LOD were imputed from a log-normal probability distribution, conditional on the value being between zero and the compound-specific LOD [42].

### Birth outcome assessment

Birth outcome measures were extracted using the hospital medical records by medical personnel. We assessed three outcomes in the analysis: (1) gestational age (continuous in weeks) defined as the duration between a self-reported date of the last menstrual period and the date of birth; (2) birth weight (continuous in grams); and (3) birth length (continuous in centimeters). We did not analyze binary classification of preterm birth or low birth weight because of the limited case numbers (*n* < 15).

### Covariates

Information on paternal and maternal characteristics was collected from prenatal baseline interviews or prenatal serum measurement. We identified potential confounders a priori based on a minimum sufficient set in a directed acyclic graph (DAG) (Figure S2), guided by the literature on the association of each with PFAS exposure and birth outcomes. Key confounders included study region (Greenland, Poland, Ukraine), maternal age at conception (continuous in years), parental age difference (continuous in years), maternal pre-pregnancy BMI (continuous in kilograms per square meter), paternal pre-conception BMI (continuous in kilograms per square meter), maternal education level (no postsecondary, postsecondary), parity (primiparous, multiparous), and maternal pregnancy serum cotinine levels (log10-transformed, nanograms per milliliter. In addition, we considered newborn sex (male, female) as a precision variable. Pregnancy serum concentration of the same PFAS exposure in the other parent (per interquartile range [IQR], nanograms per milliliter) was also adjusted as a proxy for unmeasured and shared confounders that affect both parental PFAS (see the DAG in Figure S2). Paternal education information was not included due to the large amount of missing data (*n* = 292).

### Statistical analysis

A multiple imputation strategy (5 imputations with 50 iterations) [48] that included all PFAS and the covariates was used to impute missing data for paternal and maternal characteristics based on the *mice* R package. Results from subsequent analyses were summarized using Rubin’s rule [49]. Statistical analyses were performed using R (version 4.3.0; R Development Core Team).

We first examined the distributions of paternal and maternal serum PFAS concentrations. To estimate parent-specific associations, we used multivariable linear regression models to estimate the mean differences and 95% confidence intervals (CIs) in each of the continuous birth outcomes according to per IQR increase of serum-PFAS concentrations, controlling for parental co-exposures and confounders. Log-transformation of PFAS concentrations did not improve model fit [50], thus, we used the continuous exposure model with IQR scaling based on the combined samples (Table S1). To account for the heteroscedasticity of residuals detected in our regression models, we applied the weighted least squares method, which places more weights on the data points with smaller error variance, allowing for more accurate results [51]. A square term of parental age difference was added to the model to improve model fit and allow for its non-linear relationships with the birth outcomes. Maternal serum cotinine levels were only adjusted in country-specific analyses due to multicollinearity issues when entering the country indicator in pooled analyses. Additionally adjusting for paternal serum cotinine levels did not change the results in our study; thus, paternal serum cotinine was not kept in the primary model. We used the quantile g-computation linear model approach [52] with 200 bootstrap iterations to estimate the joint mixture effects of multiple PFAS exposures on each of the birth outcomes based on the *qgcomp* package. Effect modification by newborn sex and study region were investigated in stratified analyses, and by introducing product interaction terms with the PFAS exposure in the models [42].

We conducted several additional analyses. First, we performed quantile regression to study whether the average exposure-outcome associations differed on the lower range of the outcome distribution. We used the *quantreg* package to analyze the exposure-outcome association at the small (10th) percentile of the birth outcomes and computed the standard errors with 200 bootstrap iterations. Also, to account for the influence of gestational age on birth weight and birth size measures, we performed the abovementioned primary analyses adjusting for gestational age in the model or restricted to term births only. Also, we applied generalized additive models with integrated smoothness estimation to explore potential non-linear relationships between serum PFAS concentrations and continuous birth outcomes using the *mgcv* package [53]. We obtained *p* value for non-linearity for the smooth term in the generalized additive model.

## Results

**Fig. 1:**
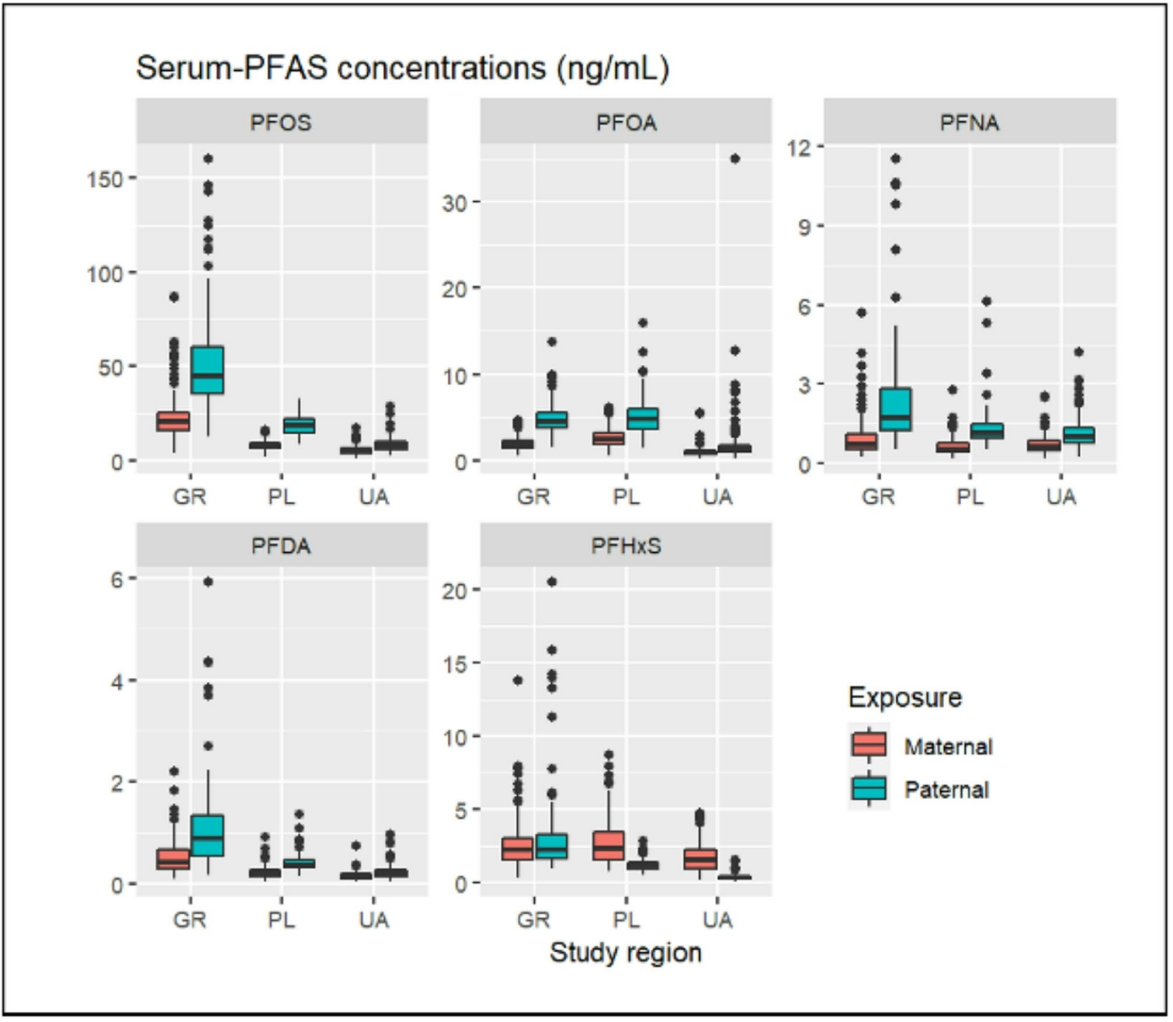
Paternal and maternal serum-PFAS concentrations (ng/mL) in the INUENDO cohort (*N* = 498). Abbreviations: GR, Greenland; PL, Poland; UA, Ukraine. In this study population, blood samples were collected during mid to late pregnancy

Table 1 shows the paternal, maternal, and newborn characteristics of the study population in each study region, separately and combined. Paternal serum-PFAS concentrations were generally approximately twice as high as maternal serum-PFAS concentrations across all three regions (Fig. 1). Among the five PFAS investigated in this study, PFOS was the highest in both parents. Parents from Greenland had the highest PFAS exposure levels during pregnancy, followed by Poland and Ukraine (Table S1). The Spearman’s correlations among the five PFAS were generally stronger in fathers than mothers (*r* = 0.40–0.92 for paternal PFAS and 0.16–0.78 for maternal PFAS). PFNA and PFDA were moderately correlated between parents in Greenland and Poland (*r* = 0.39–0.55), while the parent-paired correlations were weaker for other PFAS and for Ukrainian parents (Figure S3).

**Table 1:** Characteristics of the INUENDO cohort in Greenland, Poland, and Ukraine (*N* = 498)

Variables, categorical or continuous Median (IQR) or *n* (%) ^a^	Combined (*N* = 498)	Greenland (*N* = 178)	Poland (*N* = 142)	Ukraine (*N* = 178)
**Newborn characteristics**				
Male	259 (52.0)	98 (55.1)	67 (47.2)	94 (52.8)
Gestational age at birth, weeks	39.0 (39.0, 40.0)	40.0 (39.0, 41.0)	39.5 (38.3, 40.0)	39.0 (39.0, 40.0)
Birth weight, gram	3480 (3170, 3784)	3610 (3225, 4040)	3485 (3270, 3760)	3400 (3000, 3550)
Birth length, cm	52.0 (51.0, 54.0)	52.0 (51.0, 53.0)	54.0 (53.0, 56.0)	51.0 (50.0, 52.0)
**Maternal characteristics**				
Parity = 0	317 (64.7)	50 (28.2)	132 (93.0)	135 (78.9)
Maternal age at conception, years	26.1 (22.4, 29.8)	25.6 (22.0, 32.3)	28.0 (26.0, 30.2)	24.0 (21.0, 27.0)
Maternal pre-pregnancy BMI, kg/m^2^	22.0 (20.2, 24.8)	23.8 (21.5, 26.6)	21.0 (19.7, 22.5)	21.6 (19.9, 24.2)
Maternal education				
No postsecondary	167 (36.5)	72 (44.4)	5 (3.6)	90 (58.1)
Postsecondary	290 (63.5)	90 (55.6)	135 (96.4)	65 (41.9)
Maternal pregnancy serum-cotinine concentration, ng/mL	0.3 (0.0, 5.5)	9.1 (0.4, 90.2)	0.0 (0.0, 0.1)	0.3 (0.1, 1.5)
**Paternal characteristics**				
Paternal age at conception, years	28.7 (24.7, 33.5)	31.3 (26.1, 36.2)	29.6 (27.5, 32.6)	24.8 (22.8, 29.1)
Paternal pre-conception BMI, kg/m^2^	24.9 (23.1, 27.4)	25.5 (23.7, 28.2)	25.5 (24.0, 27.7)	23.8 (22.4, 25.8)

Abbreviation: IQR, interquartile range; BMI, body mass index

^a^Percentages of categorical variables may not sum to 100% due to rounding. Pseudo median and IQR are presented to secure participants’ anonymity. All variables had < 5% missing data except for birth length and maternal education with up to 10% missing

**Fig. 2:**
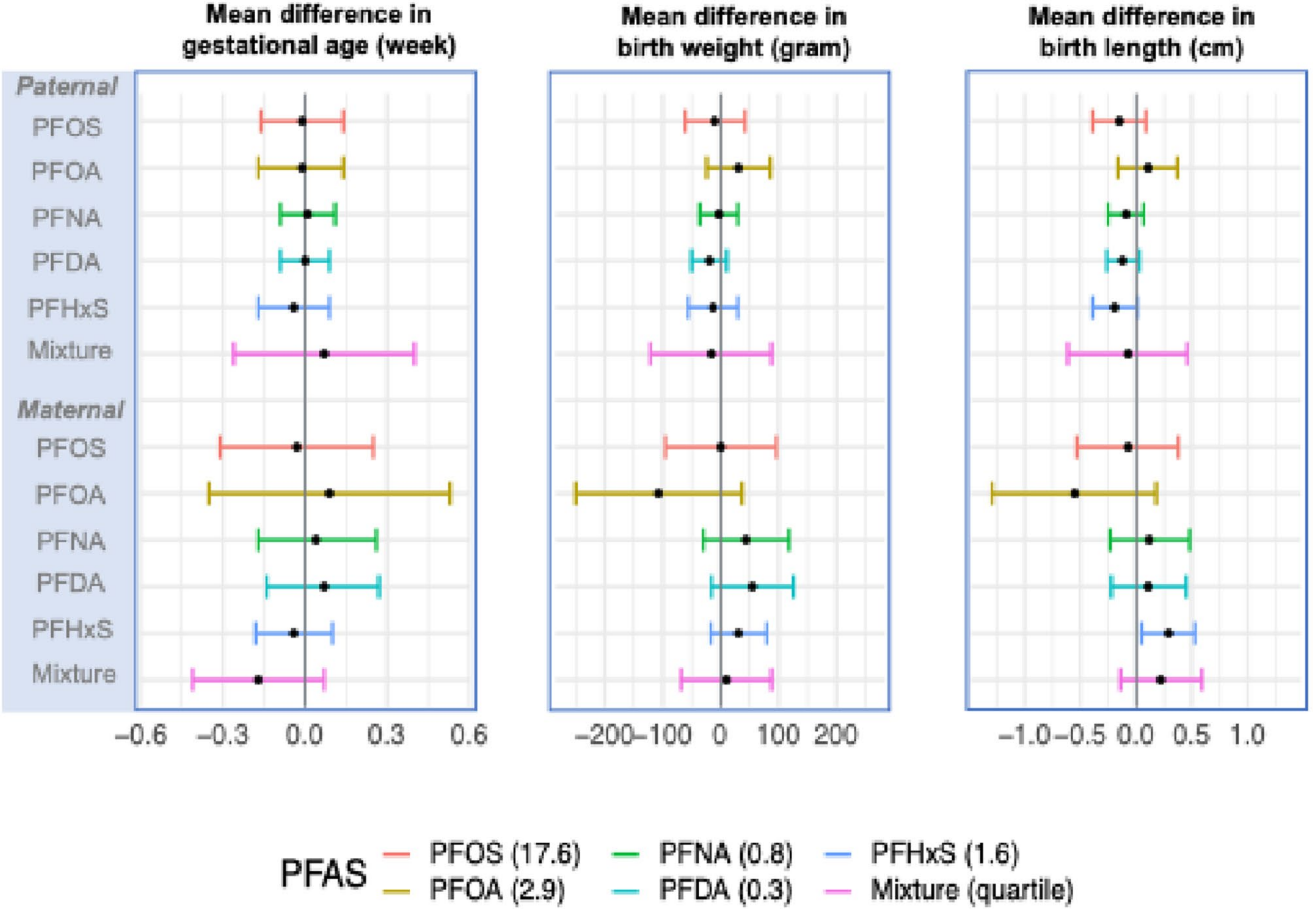
Associations of parental serum-PFAS concentrations with gestational age, birth weight, and birth length. Mean differences and 95% confidence intervals are presented. Chemical-specific associations were estimated by weighted least squares linear regression for per one interquartile range (IQR) increase in serum-PFAS concentrations (ng/mL), with IQR for each PFAS provided in parentheses. The chemical-specific associations were adjusted for study region, maternal age, parental age difference, paternal and maternal pre-pregnancy BMI, maternal education level, parity, newborn sex, and spousal exposure to the same type of PFAS. Joint associations were estimated by quantile g-computation, adjusted for the same set of covariates and spousal exposure to the same types of PFAS; PFAS mixture modeled as per one quartile increase of individual PFAS exposures of this parent

Fig. 2 presents co-adjusted parent-specific linear associations of prenatal PFAS exposures with the three birth outcomes using the combined INUENDO cohort. Overall, there were no associations found for any of the paternal or maternal PFAS exposure evaluated with gestational age. For birth weight, each IQR increase of maternal serum PFOA concentrations (a 2.9 ng/mL increase) was imprecisely associated with -107g (95% CI: -250, 37) lower birth weight, adjusted for paternal PFOA. Three paternal serum PFAS (PFNA, PFDA, PFHxS) tended to be inversely associated with birth weight, though the 95% CI included the null. For birth length, the same three paternal serum PFAS (PFNA, PFDA, PFHxS), and less clearly, PFOS, were again tentatively associated with reduced birth length (e.g., -0.09cm for PFNA, 95% CI: -0.25, 0.07; -0.19 cm for PFHxS, 95% CI: -0.39, 0.01), conditioning on maternal exposure to the same PFAS compound. However, all the 95% CIs included the null. In contrast, maternal PFOA was potentially inversely associated with birth length but the association between maternal PFHxS and birth length was positive. In the quantile g-computation analysis, no evidence suggested mixture effects of maternal or paternal exposure to multiple PFAS when co-adjusting for the same set of PFAS in the other parent (Fig. 2). 

No consistent differences appeared in newborn sex-specific analyses (Table S2). The suggestive inverse associations of maternal PFOA and paternal PFAS with birth length mostly appeared in the Greenlandic population, though *p* for interaction > 0.05 (Table S3). There were no apparent associations between PFAS and birth outcomes when in the 10th quantile of outcome distributions (Table S4). However, a per 2.9 ng/mL increase of maternal serum PFOA was associated with a decrease of 1.06 cm (95% CI: -1.91, -0.22) at the 10th percentile of birth length. After controlling for gestational age and excluding preterm-born infants (*n* = 13), the associations of paternal and maternal PFAS with birth weight and birth length persisted and exhibited an improvement in precision (Table S5). There was no evidence of non-linearity for both maternal and paternal PFAS exposures and the birth outcomes in the countries combined or in Greenland (*p* for non-linearity > 0.10). The results from crude models and models with or without adjusting for the spousal PFAS can be found in Table S6. The effect estimates did not markedly change comparing the models with or without co-adjusting for the spousal PFAS.

## Discussion

In this multi-country birth cohort of paternal and maternal serum PFAS during pregnancy, we found no associations for any parental PFAS exposure on gestational age. Some parent-specific associations were noted for offspring with lower birth weight or shorter birth length in relation to higher maternal serum PFOA or paternal serum PFOS, PFNA, PFDA, and PFHxS. However, these findings are not conclusive and require further investigation.

The collective evidence from previous meta-analyses and systematic reviews have suggested that maternal prenatal exposure to multiple PFAS compounds may adversely affect fetal growth but with a varying magnitude of effect sizes and specific to outcome measures or exposure congeners [33, 54–57]. While maternal PFAS exposure has been linked to preterm birth risks [33], the associations were inconsistent when estimating the gestational week as a continuous measure [32, 57–65]. Compared to a previous meta-analysis of 41 studies [57], our study has estimated a comparable effect size between higher maternal PFOA and lower offspring birth weight [57] and birth length [58, 66–68]. Our results further showed that co-adjusting for paternal PFAS did not markedly change the maternal PFOA effect estimate. Our mixture analyses did not reveal apparent joint exposure effects for the birth outcomes examined, suggesting the importance of evaluating compound-specific effects for adverse birth outcomes.

Concerning paternal PFAS exposure, the LIFE study conducted in Michigan and Texas was the first to report that maternal perfluorooctanesulfonamide (PFOSA) was associated with mother-reported smaller birth weight in male newborns and no paternal-offspring associations were found. Next, a Chinese pregnancy cohort study analyzed parental serum PFAS within three days before delivery and hospital-recorded birth weight reported suggestive inverse associations for five maternal PFAS exposures including PFOS, PFOA, PFNA, and PFDA, but no association for paternal PFAS and offspring birth weight [35]. More recently, the EARTH study conducted in Massachusetts among sub-fertile couples found that maternal preconception serum levels of PFOS, PFHxS, and the maternal PFAS mixture were negatively associated with birth weight. In contrast, paternal PFOS and PFHxS were positively associated with birth weight [36]. Our study estimated that higher exposures to maternal PFOA and several paternal PFAS were tentatively associated with offspring lower birth weight or shorter birth length. We noted that maternal and paternal PFHxS were associated with child’s birth length in a different direction, even after co-adjusting for the partner’s exposure. Differences in population characteristics, timing of PFAS assessment, and the exposure range studied, as well as measurement errors of exposure and outcome, may contribute to the differences in findings. In our study, co-adjusting for maternal PFAS in the model did not markedly change the results for paternal PFAS exposure. Mutual adjustment in parental association comparison could be helpful in mitigating confounding because spousal exposure may act as a proxy for unmeasured shared confounding [38, 69, 70], but bias due to multicollinearity or decreased statistical power needs attention [71]. To date, no firm conclusions can be drawn yet based on the few research findings concerning paternal PFAS exposure and limited offspring outcomes that were examined.

The present study has some strengths. First, this is the largest multi-country prospective cohort study to evaluate the associations between parental pregnancy serum PFAS and birth outcomes. The parental serum samples were collected and processed under the same conditions, ensuring that any measurement errors of exposure if any would be systematic across study sites and in the parental comparisons. Also, our outcome assessment was based on medical records with abstraction performed by trained clinical staff, minimizing outcome misclassification from self-report. Finally, we utilized mixture models to study the importance of PFAS co-exposure effects.

We also acknowledge some limitations. First, our study did not have sufficient sample sizes to evaluate preterm birth or low birth weight, which might have stronger associations with maternal prenatal PFAS exposure reported in previous studies. Secondly, paternal exposure effects on offspring health might be smaller in magnitude compared to effects from maternal intrauterine exposure. Therefore, a much larger sample size might be necessary to detect a small to moderate effect concerning paternal exposure. The paternal exposure effect may be more evident among individuals with impaired sperm function or epigenetic alterations [18–21], but such a hypothesis was not tested in our study. Co-adjusting for the spousal PFAS level in the model may somewhat address shared confounding in the family, but non-shared confounding cannot be ruled out [27, 28, 72, 73]. In addition, conditioning on gestational age may cause collider bias when there are uncontrolled common causes of gestational age and birth size [74], however, the impact may be minimal in this study considering the null associations between PFAS exposures and gestational age [75–77]. Lastly, this study did not include emerging PFAS, fluoro-alternatives, and other types of EDCs. 

## Conclusions 

This prospective multi-country cohort study provides new findings concerning parental PFAS exposure and offspring birth outcomes. Some suggestive parent-specific associations were noted for offspring lower birth weight or shorter birth length in relation to higher maternal serum PFOA or paternal serum PFOS, PFNA, PFDA, and PFHxS, but these results require further investigation. 

## Electronic supplementary material

Below is the link to the electronic supplementary material.

Supplementary Material 1

## Data Availability

To protect study participant privacy, the data cannot be shared openly. Restricted data access may be possible upon request with permission of the INUENDO team.

## Abbreviations

EDCs: Endocrine-disrupting chemicals
PFAS: Perfluoroalkyl and polyfluoroalkyl substances
US: United States
LC/MS/MS: Liquid chromatography tandem mass spectrometry
PFOS: Perfluorooctanesulfonic acid
PFOA: Perfluorooctanoate
PFNA: Perfluorononanoic acid
PFDA: Perfluorodecanoic acid
PFHxS: Perfluorohexanesulfonic acid
PFUnDA: Perfluoroundecanoic acid
PFDoDA: Perfluorododecanoic acid
LOD: Limit of detection
IQR: Interquartile range
PFOSA: Perfluorooctanesulfonamide
GR: Greenland
PL: Poland
UA: Ukraine
BMI: Body mass index
DAG: Directed acyclic graph
CI: Confidence interval
