# Supplementary material for: Paternal and maternal exposures to per- and polyfluoroalkyl substances (PFAS) and birth outcomes: a multi-country cohort study
Source: Environ Health. 2025 Jul 12;24:48. doi: 10.1186/s12940-025-01199-y (PMC12255010; doi:10.1186/s12940-025-01199-y)
Supplement: Supplementary file 1 — Supplementary Material 1 [file 12940_2025_1199_MOESM1_ESM.docx]

SUPPLEMENTARY MATERIALS

Paternal and Maternal Exposures to Per- And Polyfluoroalkyl Substances (PFAS) and Birth Outcomes: A Multi-Country Cohort Study

**Table of Contents**

[Table S1. Maternal and paternal serum PFAS concentrations in the INUENDO cohort (N=498). ^a^ 2](#_Toc193201001)

[Table S2. Associations of parental serum-PFAS concentrations with birth outcomes, stratified by newborn sex. 3](#_Toc193201002)

[Table S3. Associations of parental serum-PFAS concentrations with birth outcomes, stratified by study region. 4](#_Toc193201003)

[Table S4. Changes in birth outcomes according to parental serum-PFAS concentrations at the 10^th^ quantile (Q10) of outcome distributions. 5](#_Toc193201004)

[Table S5. Associations of parental serum-PFAS concentrations with birth outcomes in the full-term births (N=485). 6](#_Toc193201005)

[Table S6. Associations of parental serum-PFAS concentrations with birth outcomes from crude, and models with or without co-adjusting PFAS exposure in both parents (N=498). 7](#_Toc193201006)

[Figure S1. Flow diagram of study participant inclusion and exclusion. 8](#_Toc193201007)

[Figure S2. Directed acyclic graph of paternal and maternal prenatal PFAS exposures and birth outcomes. 9](#_Toc193201008)

[Figure S3. Correlation plot of paternal (p) and maternal (m) serum-PFAS concentrations in the INUENDO cohort (N=498). 10](#_Toc193201009)

Table S1. Maternal and paternal serum PFAS concentrations in the INUENDO cohort (N=498). ^a^

|  | **Median (IQR) of serum PFAS concentrations, ng/mL** | | | | | | | | |
| --- | --- | --- | --- | --- | --- | --- | --- | --- | --- |
|  | **Combined** | | | **Greenland** | | **Poland** | | **Ukraine** | |
|  | Overall | Maternal | Paternal | Maternal | Paternal | Maternal | Paternal | Maternal | Paternal |
|  | (N=996) | (N=498) | (N=498) | (N=178) | (N=178) | (N=142) | (N=142) | (N=178) | (N=178) |
| PFOS | 12.3 (6.6, 24.2) | 8.2 (5.5, 16.7) | 19.0 (9.1, 37.2) | 20.5 (15.8, 25.6) | 44.9 (35.6, 60.4) | 7.8 (6.3, 9.6) | 18.9 (14.4, 22.3) | 5.0 (3.9, 6.3) | 7.6 (5.6, 10.0) |
| PFOA | 2.1 (1.2, 4.1) | 1.6 (1.0, 2.4) | 3.7 (1.6, 5.2) | 1.8 (1.4, 2.3) | 4.5 (3.8, 5.5) | 2.5 (1.8, 3.2) | 4.7 (3.7, 6.0) | 1.0 (0.7, 1.1) | 1.3 (1.0, 1.8) |
| PFNA | 0.9 (0.6, 1.4) | 0.6 (0.5, 0.9) | 1.2 (0.9, 1.8) | 0.7 (0.5, 1.1) | 1.7 (1.2, 2.8) | 0.5 (0.4, 0.8) | 1.2 (0.9, 1.5) | 0.6 (0.5, 0.8) | 1.0 (0.8, 1.3) |
| PFDA | 0.3 (0.2, 0.5) | 0.2 (0.1, 0.4) | 0.4 (0.2, 0.7) | 0.4 (0.3, 0.7) | 0.9 (0.5, 1.3) | 0.2 (0.2, 0.3) | 0.4 (0.3, 0.5) | 0.1 (0.1, 0.2) | 0.2 (0.2, 0.3) |
| PFHxS | 1.6 (0.9, 2.5) | 2.0 (1.4, 2.9) | 1.2 (0.4, 1.9) | 2.3 (1.6, 3.1) | 2.2 (1.7, 3.3) | 2.4 (1.5, 3.5) | 1.2 (1.0, 1.4) | 1.5 (1.0, 2.2) | 0.3 (0.3, 0.5) |

Abbreviations: IQR, interquartile range**.**

^a^ Pseudomedians and IQR are presented to secure participants’ anonymity.

Table S2. Associations of parental serum-PFAS concentrations with birth outcomes, stratified by newborn sex.

|  | **Mean difference in gestational age, week (95% CI)** | | **Mean difference in birth weight, gram (95% CI)** | | **Mean difference in birth length, cm**  **(95% CI)** | |
| --- | --- | --- | --- | --- | --- | --- |
| PFAS level (per  interquartile range increase, ng/mL or per quartile increase for mixtures) | Male newborn  (N=259) | Female  newborn  (N=239) | Male newborn  (N=259) | Female newborn  (N=239) | Male  newborn  (N=227) | Female  newborn  (N=221) |
| **Paternal exposure** ^a^ |  |  |  |  |  |  |
| PFOS (17.6) | 0.09 (-0.15, 0.32) | -0.11 (-0.28, 0.07) | -2 (-82, 79) | -10 (-80, 60) | -0.23 (-0.64, 0.18) | -0.03 (-0.34, 0.27) |
| PFOA (2.9) | 0.14 (-0.16, 0.45) | -0.05 (-0.17, 0.07) | 132 (29, 235) | -5 (-64, 55) | 0.53 (-0.02, 1.07) | -0.03 (-0.30, 0.24) |
| PFNA (0.8) | 0.05 (-0.09, 0.20) | -0.02 (-0.13, 0.10) | -18 (-67, 31) | 12 (-34, 58) | -0.17 (-0.42, 0.08) | 0.01 (-0.19, 0.21) |
| PFDA (0.3) | 0.05 (-0.10, 0.19) | -0.03 (-0.14, 0.08) | -31 (-79, 16) | -2 (-44, 39) | -0.18 (-0.42, 0.07) | -0.02 (-0.20, 0.16) |
| PFHxS (1.6) | -0.02 (-0.25, 0.22) | -0.04 (-0.17, 0.10) | -28 (-100, 43) | -8 (-66, 51) | -0.25 (-0.61, 0.11) | -0.15 (-0.39, 0.08) |
| Paternal mixture ^b^ | 0.15 (-0.34, 0.64) | -0.25 (-0.67, 0.17) | 21 (-122, 163) | -38 (-188, 113) | 0.11 (-0.75, 0.97) | -0.21 (-0.87, 0.44) |
| **Maternal exposure** ^a^ |  |  |  |  |  |  |
| PFOS (17.6) | -0.08 (-0.48, 0.33) | 0.01 (-0.38, 0.39) | 35 (-100, 170) | -59 (-200, 82) | 0.10 (-0.59, 0.80) | -0.36 (-0.97, 0.25) |
| PFOA (2.9) | 0.14 (-0.59, 0.87) | 0.14 (-0.37, 0.64) | -90 (-322, 142) | -136 (-314, 42) | 0.23 (-1.01, 1.46) | -1.17 (-2.02, -0.32) |
| PFNA (0.8) | 0.07 (-0.24, 0.37) | -0.04 (-0.33, 0.25) | 89 (-12, 190) | -15 (-126, 95) | 0.38 (-0.14, 0.90) | -0.23 (-0.72, 0.27) |
| PFDA (0.3) | 0.02 (-0.28, 0.32) | 0.06 (-0.22, 0.35) | 130 (33, 228) | -37 (-139, 66) | 0.38 (-0.12, 0.88) | -0.24 (-0.69, 0.20) |
| PFHxS (1.6) | -0.01 (-0.20, 0.18) | -0.11 (-0.30, 0.08) | 41 (-21, 102) | 6 (-70, 82) | 0.23 (-0.10, 0.56) | 0.35 (-0.00, 0.70) |
| Maternal mixture ^b^ | -0.09 (-0.46, 0.29) | -0.14 (-0.41, 0.12) | 106 (-45, 257) | -45 (-156, 66) | 0.72 (0.06, 1.38) | -0.17 (-0.64, 0.31) |

^a^ Mean difference and 95% confidence interval estimated by weighted least squares linear regression, adjusted for study region, maternal age, parental age difference, paternal and maternal pre-pregnancy BMI, maternal education level, parity, newborn sex, and spousal exposure to the same type of PFAS.

^b^ Mean difference and 95% confidence interval estimated by quantile g-computation, adjusted for study region, maternal age, parental age difference, paternal and maternal pre-pregnancy BMI, maternal education level, parity, newborn sex, and spousal exposure to the same types of PFAS; PFAS mixture modeled as per quartile increase of all individual PFAS exposures of this parent.

Table S3. Associations of parental serum-PFAS concentrations with birth outcomes, stratified by study region.

|  | **Mean difference in gestational age, week**  **(95% CI)** | | | **Mean difference in birth weight, gram**  **(95% CI)** | | | **Mean difference in birth length, cm**  **(95% CI)** | | |
| --- | --- | --- | --- | --- | --- | --- | --- | --- | --- |
| PFAS level (per  interquartile range increase, ng/mL or per quartile increase for mixtures) | Greenland  (n=178) | Poland  (n=142) | Ukraine  (n=178) | Greenland (n=178) | Poland  (n=142) | Ukraine  (n=178) | Greenland  (n=128) | Poland  (n=142) | Ukraine  (n=178) |
| **Paternal exposure** ^a^ |  |  |  |  |  |  |  |  |  |
| PFOS (17.6) | -0.05 (-0.23, 0.13) | -0.16 (-1.00, 0.67) | 0.02 (-0.69, 0.74) | -18 (-79, 44) | -119 (-362, 125) | -84 (-360, 192) | -0.11 (-0.37, 0.16) | -0.51 (-1.86, 0.83) | -0.15 (-1.65, 1.34) |
| PFOA (2.9) | -0.28 (-0.70, 0.14) | 0.01 (-0.34, 0.35) | 0.02 (-0.13, 0.17) | 51 (-86, 187) | 21 (-80, 121) | 16 (-38, 70) | 0.41 (-0.22, 1.03) | 0.28 (-0.27, 0.83) | -0.07 (-0.40, 0.26) |
| PFNA (0.8) | -0.01 (-0.14, 0.12) | 0.21 (-0.10, 0.52) | -0.11 (-0.35, 0.13) | -25 (-69, 19) | 9 (-92, 111) | 18 (-71, 107) | -0.15 (-0.34, 0.04) | -0.03 (-0.59, 0.52) | 0.13 (-0.36, 0.62) |
| PFDA (0.3) | -0.02 (-0.13, 0.08) | 0.33 (-0.13, 0.79) | -0.09 (-0.44, 0.27) | -31 (-68, 7) | -15 (-153, 124) | -5 (-141, 131) | -0.11 (-0.27, 0.06) | 0.07 (-0.71, 0.86) | -0.14 (-0.87, 0.58) |
| PFHxS (1.6) | -0.04 (-0.21, 0.12) | -0.38 (-1.45, 0.69) | 0.84 (-0.32, 2.01) | -34 (-86, 19) | -242 (-543, 60) | 34 (-442, 510) | -0.23 (-0.45, -0.01) | -1.14 (-2.82, 0.54) | -0.15 (-2.61, 2.30) |
| Paternal mixture ^b^ | -0.05 (-0.44, 0.34) | 0 (-0.36, 0.35) | 0.02 (-0.18, 0.21) | -16 (-136, 105) | -41 (-149, 68) | 12 (-75, 98) | 0.07 (-0.51, 0.65) | -0.08 (-0.62, 0.47) | -0.10 (-0.57, 0.37) |
| **Maternal exposure** ^a^ |  |  |  |  |  |  |  |  |  |
| PFOS (17.6) | -0.01 (-0.34, 0.33) | -0.95 (-2.69, 0.80) | -0.48 (-1.73, 0.76) | -19 (-134, 96) | 64 (-446, 574) | 271 (-191, 732) | -0.09 (-0.58, 0.39) | 1.31 (-1.55, 4.18) | 1.03 (-1.53, 3.58) |
| PFOA (2.9) | 0.36 (-0.48, 1.20) | -0.12 (-0.82, 0.58) | -0.00 (-0.73, 0.73) | -244 (-553, 65) | -67 (-268, 133) | -20 (-317, 277) | -1.01 (-2.36, 0.34) | -1.00 (-2.12, 0.12) | 0.00 (-1.55, 1.56) |
| PFNA (0.8) | 0.09 (-0.23, 0.41) | -0.19 (-0.84, 0.46) | 0.02 (-0.33, 0.37) | 64 (-42, 171) | 69 (-129, 267) | 82 (-49, 213) | 0.18 (-0.28, 0.63) | 0.00 (-1.09, 1.09) | 0.54 (-0.21, 1.29) |
| PFDA (0.3) | 0.10 (-0.15, 0.36) | -0.02 (-0.70, 0.66) | -0.14 (-0.71, 0.44) | 67 (-19, 154) | 140 (-56, 336) | 49 (-168, 266) | 0.09 (-0.29, 0.47) | 0.27 (-0.84, 1.39) | 0.70 (-0.50, 1.90) |
| PFHxS (1.6) | 0.05 (-0.16, 0.26) | -0.19 (-0.45, 0.08) | -0.16 (-0.42, 0.09) | 67 (-6, 140) | -30 (-105, 44) | 5 (-95, 104) | 0.22 (-0.13, 0.58) | -0.06 (-0.47, 0.36) | 0.88 (0.33, 1.43) |
| Maternal mixture ^b^ | 0.05 (-0.30, 0.41) | -0.19 (-0.56, 0.19) | -0.10 (-0.33, 0.14) | -8 (-123, 108) | -2 (-112, 108) | 41 (-42, 125) | -0.15 (-0.62, 0.32) | 0.10 (-0.48, 0.68) | 0.69 (0.27, 1.12) |

^a^ Mean difference and 95% confidence interval estimated by weighted least squares linear regression, adjusted for maternal age, parental age difference, paternal and maternal pre-pregnancy BMI, maternal education level, parity, newborn sex, maternal serum-cotinine concentration, and spousal exposure to the same type of PFAS.

^b^ Mean difference and 95% confidence interval estimated by quantile g-computation, adjusted for maternal age, parental age difference, paternal and maternal pre-pregnancy BMI, maternal education level, parity, newborn sex, spousal exposure to the same types of PFAS, and maternal serum-cotinine concentration; PFAS mixture modeled as per quartile increase of all individual PFAS exposures of this parent.

Table S4. Changes in birth outcomes according to parental serum-PFAS concentrations at the 10^th^ quantile (Q10) of outcome distributions.

|  | **Change in gestational age, week (95% CI)** | **Change in birth weight, gram (95% CI)** | **Change in birth length, cm (95% CI)** |
| --- | --- | --- | --- |
| Per interquartile range increase in PFAS | Overall  (N=498) | Overall  (N=498) | Overall  (N =448) |
| **Paternal exposure** ^a^ |  |  |  |
| PFOS | 0.18 (-0.07, 0.43) | 79 (-17, 175) | 0.08 (-0.40, 0.56) |
| PFOA | 0.05 (-0.21, 0.32) | 49 (-59, 157) | 0.01 (-0.39, 0.41) |
| PFNA | 0.13 (-0.02, 0.28) | -1 (-85, 83) | -0.11 (-0.44, 0.22) |
| PFDA | 0.07 (-0.12, 0.26) | 10 (-77, 96) | 0.02 (-0.41, 0.44) |
| PFHxS | -0.09 (-0.31, 0.13) | -34 (-102, 34) | -0.14 (-0.52, 0.25) |
| **Maternal exposure** ^a^ |  |  |  |
| PFOS | 0.19 (-0.30, 0.69) | -86 (-351, 179) | -0.66 (-1.65, 0.33) |
| PFOA | -0.03 (-0.98, 0.91) | -56 (-314, 202) | -1.06 (-1.91, -0.22) |
| PFNA | 0.19 (-0.18, 0.55) | 66 (-166, 298) | -0.04 (-0.96, 0.88) |
| PFDA | 0.30 (-0.12, 0.72) | 12 (-227, 250) | -0.30 (-1.33, 0.72) |
| PFHxS | 0.17 (-0.08, 0.41) | 90 (-7, 188) | -0.07 (-0.57, 0.44) |

Abbreviation: Q10, the 10^th^ quantile.  ^a^ Estimated by quantile regression, adjusted for study region, maternal age, parental age difference, paternal and maternal pre-pregnancy BMI, maternal education level, parity, newborn sex, and spousal exposure to the same type of PFAS.

Table S5. Associations of parental serum-PFAS concentrations with birth outcomes in the full-term births (N=485).

|  | **Mean difference in birth weight, gram (95% CI)** | **Mean difference in birth length, cm (95% CI)** |
| --- | --- | --- |
| PFAS (per  interquartile increase, ng/mL or per quartile increase for mixtures) | Overall  (N=485) | Overall  (N=437) |
| **Paternal exposure** ^a^ |  |  |
| PFOS (17.6) | -22 (-69, 24) | -0.24 (-0.46, -0.02) |
| PFOA (2.9) | 28 (-20, 77) | 0.07 (-0.16, 0.31) |
| PFNA (0.8) | -14 (-43, 16) | -0.14 (-0.29, 0.00) |
| PFDA (0.3) | -33 (-61, -6) | -0.19 (-0.32, -0.06) |
| PFHxS (1.6) | -16 (-55, 22) | -0.19 (-0.37, -0.01) |
| Paternal mixture ^b^ | -46 (-138, 46) | -0.30 (-0.76, 0.17) |
| **Maternal exposure** ^a^ |  |  |
| PFOS (17.6) | -1 (-87, 85) | -0.05 (-0.46, 0.36) |
| PFOA (2.9) | -96 (-228, 36) | -0.48 (-1.15, 0.19) |
| PFNA (0.8) | 45 (-22, 111) | 0.17 (-0.15, 0.49) |
| PFDA (0.3) | 60 (-3, 122) | 0.17 (-0.13, 0.48) |
| PFHxS (1.6) | 34 (-9, 78) | 0.26 (0.04, 0.48) |
| Maternal mixture ^b^ | 46 (-35, 128) | 0.30 (-0.05, 0.66) |

^a^ Estimated by weighted least squares linear regression, adjusted for study region, maternal age, parental age difference, paternal and maternal pre-pregnancy BMI, maternal education level, parity, newborn sex, spousal exposure to the same type of PFAS, and gestational age (continuous in weeks).

^b^ Estimated by quantile g-computation, adjusted for the study region, maternal age, parental age difference, paternal and maternal pre-pregnancy BMI, maternal education level, parity, newborn sex, spousal exposure to the same types of PFAS, and gestational age (continuous in weeks); PFAS mixture modeled as per quartile increase of all individual PFAS exposures of this parent.

Table S6. Associations of parental serum-PFAS concentrations with birth outcomes from crude, and models with or without co-adjusting PFAS exposure in both parents (N=498).

|  | **Mean difference in gestational age, week**  **(95% CI)** | | | **Mean difference in birth weight, gram**  **(95% CI)** | | | **Mean difference in birth length, cm**  **(95% CI)** | | |
| --- | --- | --- | --- | --- | --- | --- | --- | --- | --- |
| PFAS level (per  interquartile range increase, ng/mL or per quartile increase for mixtures) | Model A (crude) ^a^ | Model B (adjusted for confounders only) ^b^ | Model C (adjusted confounders and spousal PFAS) ^c^ | Model A (crude) ^a^ | Model B (adjusted for confounders only) ^b^ | Model C (adjusted confounders and spousal PFAS) ^c^ | Model A (crude) ^a^ | Model B (adjusted for confounders only) ^b^ | Model C (adjusted confounders and spousal PFAS) ^c^ |
| **Paternal exposure** |  |  |  |  |  |  |  |  |  |
| PFOS (17.6) | 0.08 (-0.02, 0.18) | -0.01 (-0.16, 0.13) | -0.01 (-0.16, 0.14) | 84 (47, 120) | -9 (-58, 41) | -10 (-61, 42) | -0.14 (-0.28, 0.01) | -0.16 (-0.39, 0.07) | -0.15 (-0.39, 0.09) |
| PFOA (2.9) | 0.07 (-0.07, 0.22) | -0.01 (-0.16, 0.14) | -0.01 (-0.17, 0.14) | 123 (70, 176) | 25 (-29, 80) | 31 (-24, 86) | 1.00 (0.70, 1.29) | 0.08 (-0.18, 0.34) | 0.11 (-0.16, 0.37) |
| PFNA (0.8) | 0.03 (-0.05, 0.12) | 0.02 (-0.07, 0.11) | 0.01 (-0.09, 0.11) | 35 (2, 67) | 4 (-25, 34) | -3 (-36, 30) | -0.13 (-0.24, -0.02) | -0.07 (-0.21, 0.07) | -0.09 (-0.25, 0.07) |
| PFDA (0.3) | 0.05 (-0.02, 0.12) | 0.02 (-0.06, 0.10) | 0.00 (-0.09, 0.09) | 43 (17, 69) | -8 (-34, 17) | -19 (-50, 11) | -0.13 (-0.20, -0.05) | -0.09 (-0.21, 0.03) | -0.12 (-0.26, 0.03) |
| PFHxS (1.6) | 0.06 (-0.07, 0.19) | -0.04 (-0.17, 0.08) | -0.04 (-0.17, 0.09) | 95 (43, 146) | -11 (-53, 32) | -13 (-56, 30) | -0.11 (-0.27, 0.04) | -0.16 (-0.36, 0.04) | -0.19 (-0.39, 0.01) |
| Paternal mixture ^d^ | 0.12 (-0.01, 0.24) | 0.07 (-0.26, 0.39) | 0.07 (-0.26, 0.40) | 125 (84, 167) | -23 (-121, 75) | -16 (-121, 88) | 0.50 (0.28, 0.72) | -0.10 (-0.62, 0.41) | -0.07 (-0.61, 0.46) |
| **Maternal exposure** |  |  |  |  |  |  |  |  |  |
| PFOS (17.6) | 0.12 (-0.08, 0.33) | -0.03 (-0.30, 0.24) | -0.03 (-0.31, 0.25) | 162 (83, 242) | -2 (-93, 89) | 1 (-95, 96) | -0.23 (-0.52, 0.06) | -0.15 (-0.58, 0.29) | -0.07 (-0.53, 0.38) |
| PFOA (2.9) | 0.25 (-0.12, 0.61) | 0.09 (-0.35, 0.52) | 0.09 (-0.35, 0.53) | 114 (-11, 238) | -96 (-237, 45) | -107 (-250, 37) | 2.53 (1.82, 3.23) | -0.52 (-1.24, 0.21) | -0.55 (-1.29, 0.18) |
| PFNA (0.8) | 0.08 (-0.07, 0.22) | 0.06 (-0.14, 0.25) | 0.04 (-0.17, 0.26) | 57 (-4, 117) | 42 (-24, 109) | 44 (-30, 118) | -0.11 (-0.39, 0.17) | 0.04 (-0.28, 0.36) | 0.12 (-0.23, 0.48) |
| PFDA (0.3) | 0.10 (-0.03, 0.24) | 0.07 (-0.10, 0.24) | 0.07 (-0.14, 0.27) | 116 (58, 174) | 39 (-22, 99) | 55 (-16, 126) | -0.10 (-0.34, 0.13) | -0.02 (-0.30, 0.26) | 0.11 (-0.22, 0.45) |
| PFHxS (1.6) | -0.02 (-0.16, 0.12) | -0.04 (-0.18, 0.10) | -0.04 (-0.18, 0.10) | 50 (5, 96) | 30 (-18, 78) | 31 (-17, 80) | 0.54 (0.27, 0.80) | 0.27 (0.03, 0.50) | 0.29 (0.05, 0.53) |
| Maternal mixture ^d^ | 0.08 (-0.08, 0.23) | -0.15 (-0.39, 0.08) | -0.17 (-0.41, 0.07) | 102 (48, 156) | 11 (-65, 88) | 10 (-68, 88) | 0.66 (0.38, 0.93) | 0.21 (-0.15, 0.57) | 0.22 (-0.14, 0.59) |

^a^ Model A (crude): chemical-specific associations were estimated by weighted least square linear regression, with no covariates in the model.

^b^ Model B (adjusted for confounders only): chemical-specific associations were estimated by weighted least square linear regression, adjusted for study region, maternal age, age difference between paternal and maternal age, paternal and maternal pre-pregnancy BMI, maternal education level, parity, and newborn sex; the model did not adjust for spousal exposure to the same type of PFAS.

^c^ Model C (adjusted confounders and spousal PFAS): chemical-specific associations were estimated by weighted least square linear regression, adjusted for study region, maternal age, age difference between paternal and maternal age, paternal and maternal pre-pregnancy BMI, maternal education level, parity, newborn sex, and spousal exposure to the same type(s) of PFAS.

^d^ Joint associations estimated by quantile g-computation. PFAS mixture was modeled as per quartile increase of all individual PFAS exposures of this parent.


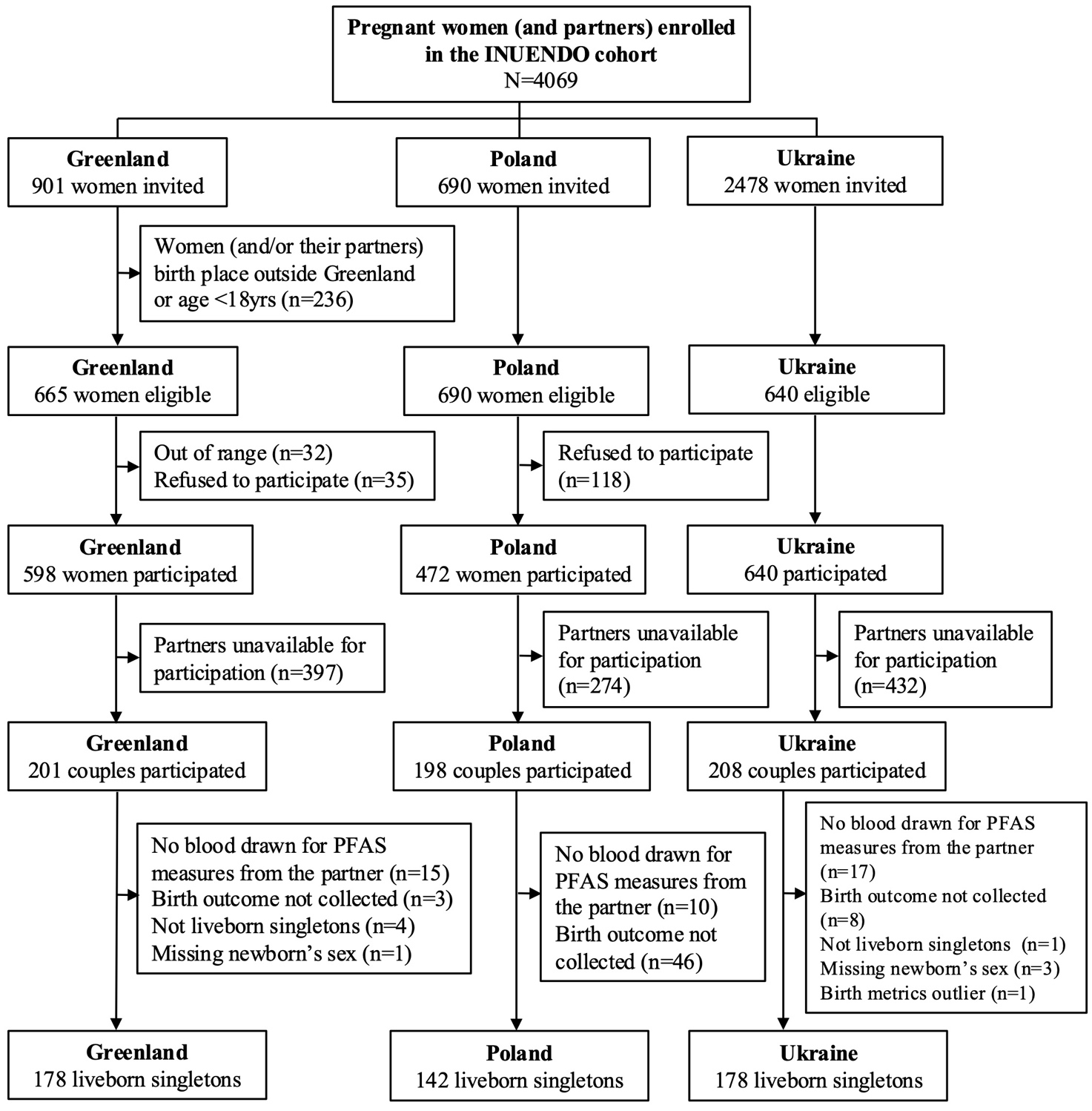


Figure S1. Flow diagram of study participant inclusion and exclusion.

**
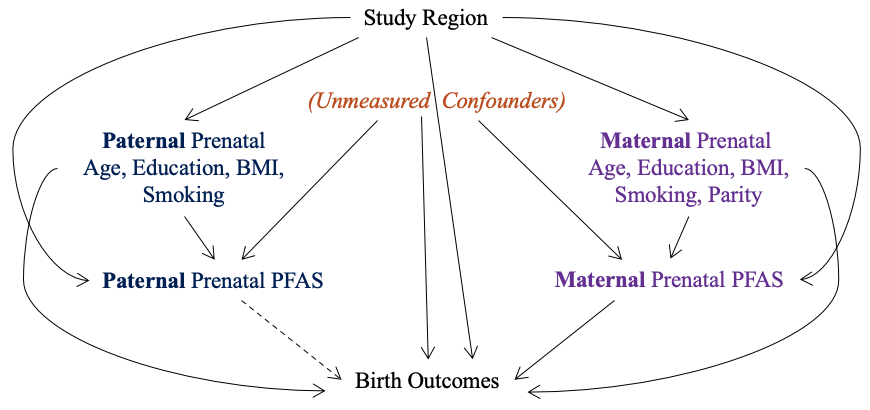
**

Figure S2. Directed acyclic graph of paternal and maternal prenatal PFAS exposures and birth outcomes. Possible interrelations between potential confounders are not shown. The dotted line indicates an unclear paternal effect.


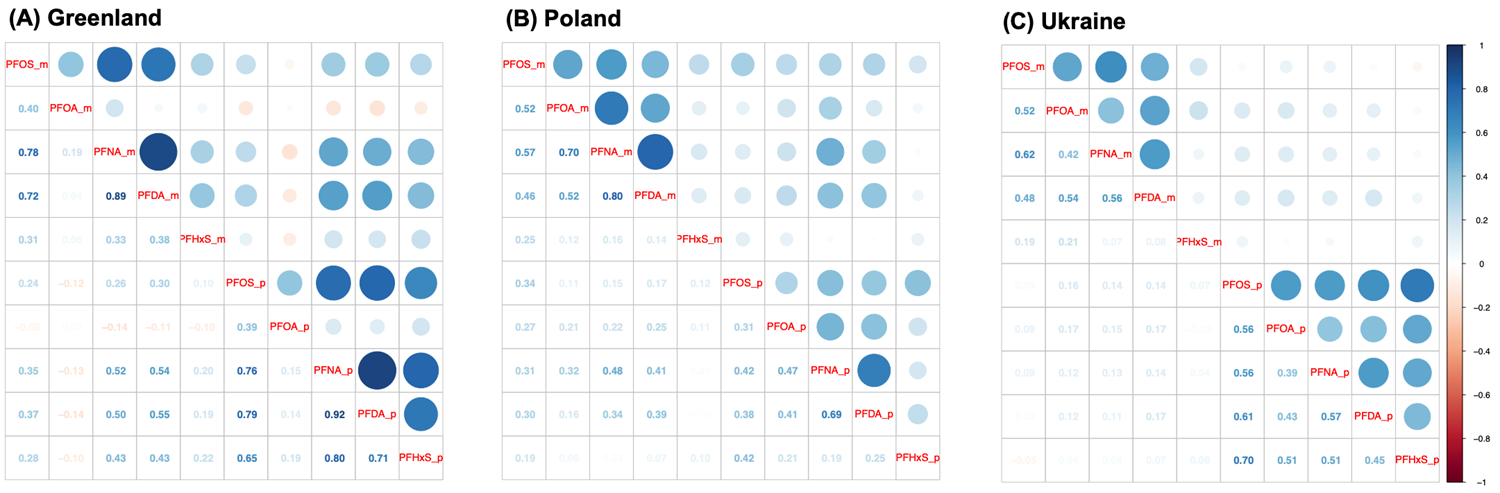


Figure S3. Correlation plot of paternal (p) and maternal (m) serum-PFAS concentrations in the INUENDO cohort (N=498). Spearman correlation coefficients are presented.
